# Outcomes of a Non‐Surgical Combination Therapy for Keloids in Skin of Color Patients: A 10‐Year Follow‐Up Study

**DOI:** 10.1111/ijd.17829

**Published:** 2025-05-05

**Authors:** Marina Patrus Ananias de Souza Brandão, Carolina Fernandes Otoni Vieira, Daniel Brasil Braga Rocha, Márcio Roberto Silva, Jussara Aparecida da Silva, Michael H. Gold, Gisele Viana de Oliveira

**Affiliations:** ^1^ Post Graduation Department Medical Sciences Faculty of Minas Gerais (FCMMG) Belo Horizonte Brazil; ^2^ EMBRAPA Gado de Leite Juiz de Fora Brazil; ^3^ Physiotherapy Department GV Dermatology Belo Horizonte MG Brazil; ^4^ Gold Skin Care Dermatology Nashville Tennessee USA; ^5^ Santa Casa de Misericórdia de MG Belo Horizonte Brazil

**Keywords:** ASAP protocol, Fitzpatrick skin phototype, intense pulsed laser therapy, keloids, recurrence, skin of color, treatment guideline

## Abstract

**Background:**

Keloid treatment remains challenging due to high recurrence rates, particularly in patients with higher phototypes. This study aimed to assess the resolution, patient satisfaction, and recurrence rates in 79 keloids treated with a sequential non‐surgical protocol combining long‐term occlusion with hydrocolloid dressings, monthly injections of triamcinolone and 5‐fluorouracil (5‐FU), and intense pulsed light therapy. This approach is expected to lower recurrence, improve resolution, and increase patient satisfaction compared to traditional treatments.

**Methods:**

This longitudinal observational study included 79 keloids in 55 patients with Fitzpatrick phototypes III or higher. Data were collected retrospectively from medical records and clinical appointments. Patients were followed for 19–123 months after completing treatment. Outcomes evaluated included keloid resolution, patient satisfaction, and recurrence rates.

**Results:**

The patients had a mean age of 39 years and underwent treatment for a median of 10 months. Pain was reported in 8.9% of cases, and only one recurrence (1.3%) occurred five years post‐treatment. Complete resolution was achieved in 82.3% of keloids, and 94.9% of patients reported maximum satisfaction.

**Conclusions:**

The sequential non‐surgical approach evaluated in this study demonstrated low recurrence and high satisfaction rates. Thus, it may represent a promising treatment option for keloid patients with higher phototypes.

## 1 Introduction

The term “keloid” is derived from the Greek word “*chele*,” which means “crab claw,” representing an allusion to its continuous growth [1]. Histologically, it is a fibrous cutaneous disorder [2] that occurs due to chronic inflammation and changes in collagen production rates in the reticular dermis [3, 4]. Keloids extend beyond the original margins and do not tend to regress [1, 2]. The occurrence of keloids is influenced by genetic factors, with a higher prevalence among Africans, Asians, and Hispanics and a lower prevalence among Whites [5]. Its high prevalence in Latin America is explained by the large admixture of races during the colonization period, which led to a wide range of skin pigmentation [5, 6, 7].

Despite the availability of several therapies to treat keloids [8], the success of each approach depends on its ability to prevent keloid recurrence [9]. The recurrence can be as high as 45%–100% after surgery alone [10], dropping to 33% following steroid injections within only 1 year of follow‐up [11]. Bleomycin injections present a 50% recurrence rate after 18 months [12], and verapamil a 20.9% recurrence rate after 3 months [13]. This underscores a significant gap in the availability of truly effective therapeutic modalities for keloid treatment, characterized by low recurrence rates and sustained efficacy over extended follow‐up periods. The ASAP algorithm (an acronym that stands for A—Assessment, S—Softening, A—Application of technologies and antifibrotic drugs, P—Pigmentation) proposes initiating keloid treatment with occlusion using hydrocolloid dressings, which improve the pliability of keloids (Table 1) [14, 15]. The occlusion is maintained until the complete flattening of the keloid. A previous study [16] showed that occlusions soften the keloids, enabling less painful injections of a combination of 5‐fluorouracil (5‐FU) and triamcinolone [17]. This combination is administered in small doses during monthly sessions: 0.2 mL of a 1:1 (5‐FU: triamcinolone) solution for 1 cm of the keloid. When used immediately before injections, intense pulsed light (IPL) therapy targets the vascularity of keloids and induces immediate edema, thereby causing additional softening [7, 14, 15]. IPL sessions and injections are maintained until the improvement of erythema. This method has been employed in public outpatient clinics and private practice [7, 14, 15].

**TABLE 1:** The ASAP method to treat keloids.

A. Assessment	In the first appointment, assess and document the keloid. Avoid premature steroid injections in very stiff and hard lesions
S. Soften the keloid before injections	Long‐term use of hydrocolloid dressings leads to softening of the keloid stiffness. The dressings are kept in place covering the whole keloid and changed each 2 weeks, until their complete softening and flattening
A. Approach the keloid using technologies	Each month, patients return to appointments—the dressings are removed, skin is cleansed and keloids that have been previously softened receive low doses of a combination of triamcinolone and 5‐FU (1:1). Immediately before the injection, keloids are treated with IPL, that leads to an immediate edema (softening) also helping the injections. In this study, only IPL was used every 1–2 months
P. Treat pigmentation	Using topical tretinoin, performing retinoic acid peels and technologies (the keloids of the current study only received topical retinoids and retinoic acid peels)

Abbreviations: 5‐FU, 5‐fluorouracil; IPL, intense pulsed light.

The study was motivated by the scarcity of research focused on effective combined treatments for keloids that demonstrate low recurrence rates with a long‐term follow‐up. In response to this gap, this study aimed to evaluate the recurrence rates of keloids managed with a combined therapeutic regimen incorporating hydrocolloid dressings, 5‐FU/triamcinolone injections, and IPL therapy in patients with higher phototypes.

## 2 Materials and Methods

This longitudinal, retrospective observational study was approved by the institutional ethical committee and registered in Plataforma Brazil (#6.674.720—CAAE 65135922.0.3001.5134). The study followed the Strengthening the Reporting of Observational Studies in Epidemiology (STROBE) guidelines and was registered at ClinicalTrials.gov with the protocol number 6.647.720. Informed consent was obtained from all participants. The inclusion criteria of this study specified male and female patients aged 18–75 years with a clinical diagnosis of a keloid who had completed their treatment at least 19 months before the initiation of the study. Patients were excluded if they had undergone radiotherapy and had phototypes I‐II according to Fitzpatrick's scale. All participants were treated in a public outpatient clinic, as well as in a private clinic in Belo Horizonte, Brazil.

All patients received the ASAP treatment, which included the placement of hydrocolloid dressings for at least 2 months, changed every 15 days, 5‐FU (50 mg/mL) and triamcinolone hexacetonide (20 mg/mL) monthly injections in a 1:1 proportion (0.2 mL per session, after the placement of dressings), and IPL sessions (Etherea, Filter 540, energy 12‐18 J, Pulse 5–15 μs) before each injection. Treatment was maintained until the flattening of the lesion and the improvement of erythema were achieved.

Data were collected retrospectively over time by analyzing past medical records and during clinical appointments (telemedicine and face‐to‐face interviews) at least 19 months after treatment completion.

Keloid recurrence was assessed using the comparison of images taken before and after treatment completion, requiring evidence of increased erythema and lesion thickness compared to the baseline presentation. This evaluation was conducted by the same independent observer who was not involved in the study.

Patients reported treatment satisfaction using a five‐point Likert scale [18], where a score of 5 was equivalent to the patient being totally satisfied; 4 indicated the patient was partially satisfied; 3 indicated the patient was moderately satisfied; 2 indicated the patient was slightly satisfied; and 1 indicated the patient was very dissatisfied.

Resolution was the primary outcome, defined as the complete flattening of the keloid, and it was assessed using a five‐point scale. A score of 5 indicated both erythema improvement and complete lesion flattening. A score of 4 represented significant improvement, characterized by complete lesion flattening with residual erythema. A score of 3 denoted moderate improvement, with a reduction in erythema and partial lesion flattening, though the keloid remained elevated. A score of 2 indicated mild improvement, with slight erythema reduction and minimal flattening. Finally, a score of 1 reflected no improvement, with no observable changes in erythema or lesion thickness.

Keloids were also rated using the Detroit scale [19]. The questionnaire was completed by the patient before and after treatment and by an independent observer who was not in charge of the treatment. The same observer rated all patients.

### 2.1 Statistical Analysis

A series of descriptive and correlational analyses was carried out as essential components of the data summarization and evaluation process. Primarily, descriptive assessments were conducted to synthesize demographic and main clinical parameters. We reported continuous variables (age, duration of keloid, and duration of treatment) as mean ± standard deviation (SD) or median (interquartile range, IQR), based on inherent data distribution. Additionally, categorical variables (gender, anatomical region of keloid, recurrence, side effects, condition resolution, and treatment satisfaction) were reported as frequencies and percentages (%). We also evaluated potential associations between categorical variables using the chi‐square test or Fisher's exact test, as deemed appropriate. Based on data normality, an independent t‐test or Mann–Whitney U test was applied to identify differences in continuous variables among groups. The association between treatment duration and keloid resolution was evaluated using linear regression, while standard logistic regression models were utilized to evaluate potential predictors of recurrence and resolution of keloid. To evaluate the monotonic relationship between keloid evolution time (in months) and treatment duration until keloid resolution (in months), we performed a Spearman's rank correlation test. Additionally, we evaluated the association between these two variables across percentiles of the treatment duration distribution. We selected this approach to better capture existing differences in the association between the time of evolution of keloid and treatment duration with “shorter” (τ = 0.25), “median” (τ = 0.50), and “longer” (τ = 0.75) treatment durations. We established a statistical significance at *p* < 0.05. All statistical assessments were carried out using R Studio (version 4.4.1).

## 3 Results

### 3.1 Demographics and Clinical Characteristics

**FIGURE 1:**
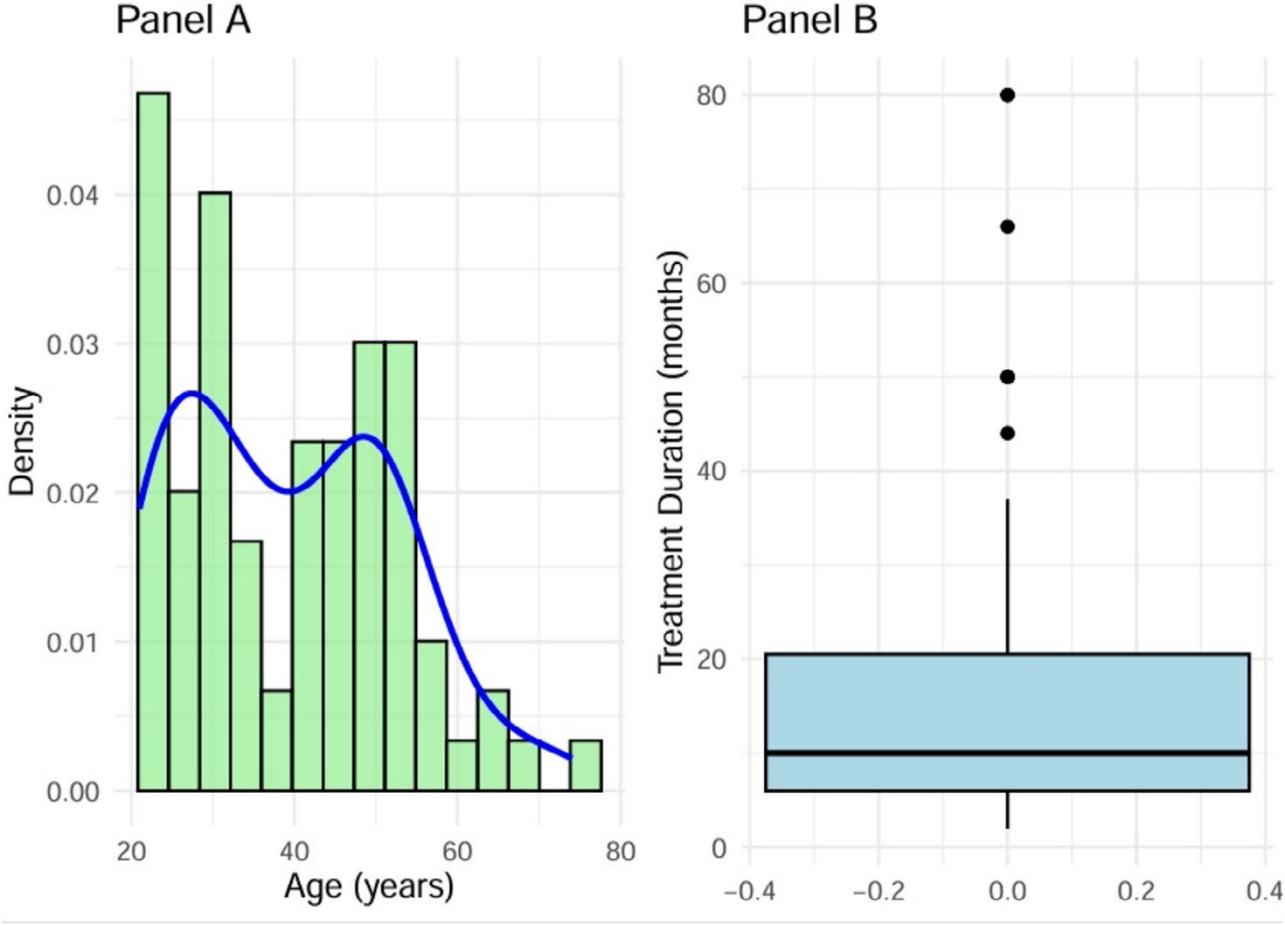
Patient age (Panel A) and treatment duration distribution (Panel B). Mean age: 39.46 ± 13.28 years (range: 21–74 years), mean treatment duration: 18.95 ± 20.65 months (median time: 10 months, interquartile range: 6–20.5 months).

Our study enrolled a total of 79 patients, with a mean age of 39.46 ± 13.28 years (range: 21–74 years). Most of the included patients were female (78.5%). Regarding treatment duration, the mean was 18.95 ± 20.65 months (median time of 10 months, IQR: 6–20.5 months). The distribution of patient age (Panel A) and treatment duration (Panel B) is shown in Figure 1. We included patients with lesions in different anatomical regions, including on the anterior trunk (27.8%), abdomen/pelvis (19.0% each), and posterior trunk and limbs (11.4%). A minor number of patients developed hypertrophic healing on the head/neck (8.9%) or other anatomical regions (2.5%). The factors that led to the development of keloids were surgical procedures (35.4%), sharp or blunt force trauma (27.8%), burns (7.9%), acne (11.4%), infection (7.6%), and spontaneous appearance (5%). The only side effect reported by the patients during the treatment was pain in 7 (8.9%) keloids, whereas 59 keloids showed a complete improvement in symptoms (pain and itching) within 4 months of treatment initiation. A significant improvement was observed before and after the treatment. The Detroit scores significantly differed among the patients (*p* < 0.001) and were equal to 6.12 (95% CI, 5.56–6.69).

### 3.2 Clinical Outcomes: Recurrence, Resolution, and Satisfaction

**FIGURE 2:**
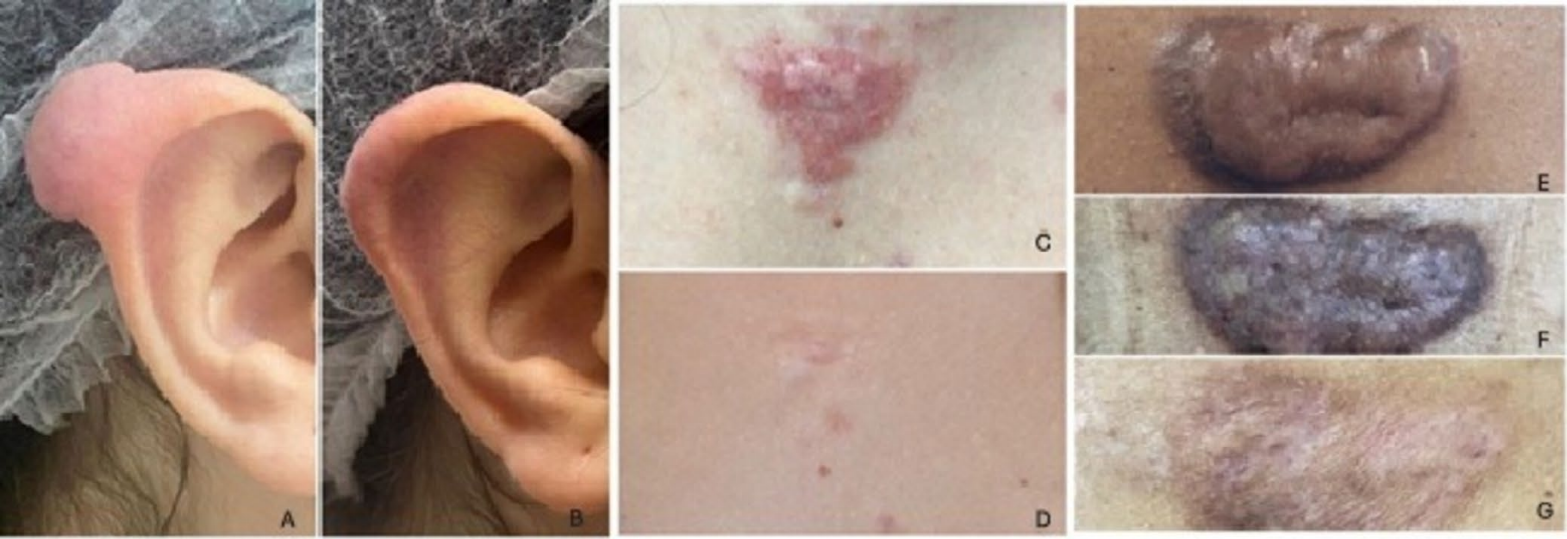
Ear keloid before (A) and after (B) treatment. Chest keloid before (C) and after (D) treatment: Complete resolution. Chest keloid before (E) and after 2 months of treatment (F); final outcome (G).

As far as treatment outcomes are concerned, we observed a minimal recurrence rate among treated individuals (1.3%), and 65 patients (82.3%) achieved complete keloid resolution, which included improvement of erythema and complete flattening of the lesion. Maximum satisfaction scores (five points on the Likert scale) were achieved in 75 lesions (94.9%) (Table 2). According to our statistical assessments of association, no significant association between gender and recurrence (*p* = 0.4855), nor between gender and complete resolution (*p* = 0.2863), was identified. The images shown in Figure 2 depict before and after images.

**TABLE 2:** Recurrence, resolution, and satisfaction rates of all patients.

Variable	Total (%)
Recurrence (minimum follow‐up of 19 months)	
Yes	1 (1.3)
No	78 (98.7)
Resolution	
Total resolution (5/5) Improvement of erythema and complete flattening	65 (82.3)
Partial resolution (4/5) Residual erythema and complete flattening	14 (17.7)
Reasonable resolution (3/5) Reduction in erythema and partial flattening	0 (0)
Slight resolution (2/5) Slight erythema reduction and minimal flattening	0 (0)
No resolution (1/5) No observable changes in erythema or thickness	0 (0)
Satisfaction with the final outcome	
Very satisfied (5/5)	75 (94.9)
Satisfied (4/5)	4 (5.1)
Neutral (3/5)	0 (0)
Dissatisfied (2/5)	0 (0)
Very dissatisfied (1/5)	0 (0)

### 3.3 Statistical Analysis and Predictors of Resolution

Throughout a linear regression analysis, our findings revealed no relevant correlation between treatment duration and the probability of reaching a complete resolution status (*p* = 1.00). Moreover, our logistic regression observations failed to identify treatment duration as a relevant predictor for full resolution (OR = 0.999, *p* = 1.00). This reinforces the concept that the length of treatment itself might not be considered an essential determinant of clinical success. Nevertheless, we observed that adverse effects retrieved significant association results (lower likelihood of complete keloid resolution, *p* = 0.019). Our assessments failed to identify any significant association between keloid location and recurrence (*p* = 0.8543), complete resolution (*p* = 0.2443), adverse effects (*p* = 0.5253), or treatment duration (*p* = 0.247).

Patient satisfaction was additionally analyzed concerning different primary outcomes. Interestingly, we identified that higher satisfaction rates were positively correlated with complete keloid resolution (*p* = 0.000176). Notwithstanding, no significant correlation was identified with patient satisfaction and recurrence (*p* = 1.00) or reporting/occurrence of adverse events attributed to the delivered intervention (*p* = 0.7927).

We explored potential predictors of recurrence and resolution using a multivariate logistic regression model. Nevertheless, we identified that the model evaluating recurrence did not converge properly, potentially due to the low recurrence rate among patients (1.3%), which limits the statistical power. Likewise, the logistic regression assessing complete resolution that we performed did not identify relevant predictors, indicating that the analyzed clinical and demographic features did not provide sufficient explanation for the variability in primary treatment outcomes.

**FIGURE 3:**
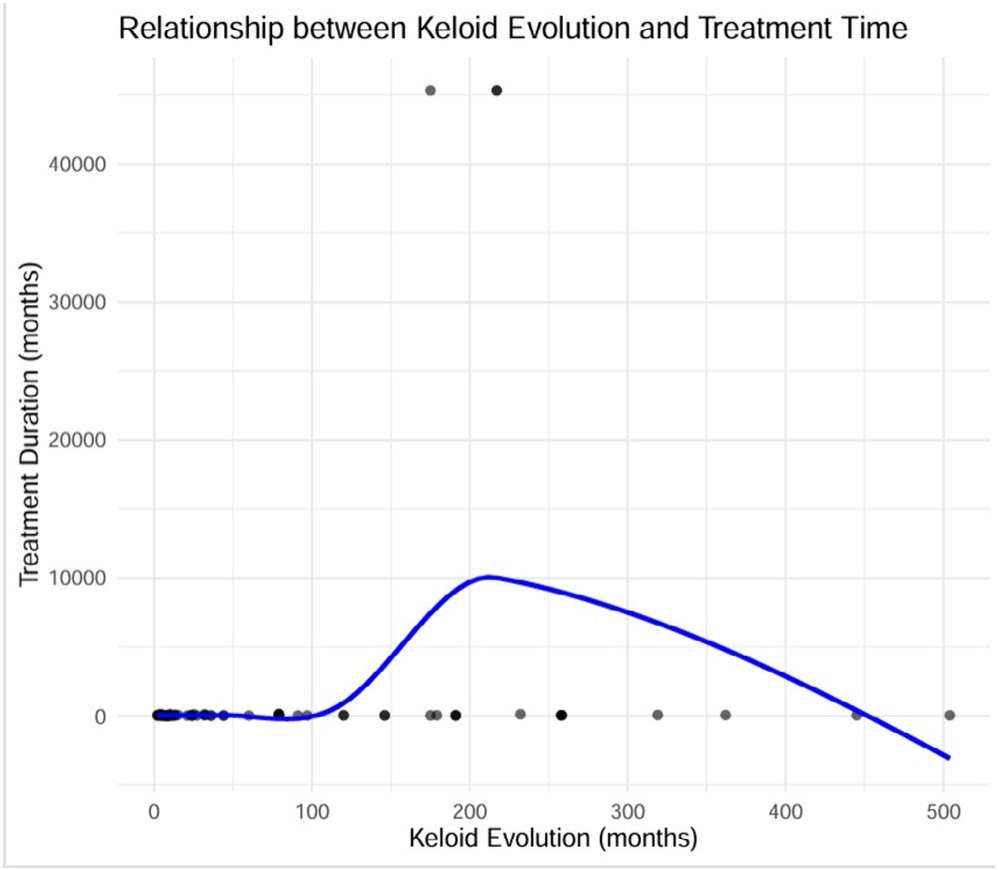
Association between keloid evolution and treatment duration (in months). Assessment showing a statistically significant correlation between keloid evolution time and treatment (rho = 0.26, *p* = 0.0186), although with a weak association.

We evaluated the influence of several potential effect modulators on treatment duration (in months). Additionally, our results showed that there is a significant difference in treatment duration among individuals who had received corticoid infiltration before ASAP treatment and those who had not (W = 253.5, *p* = 0.0022), evidencing that prior corticosteroid infiltration is associated with longer treatment duration. As far as the correlation between keloid evolution time and treatment duration is concerned, our assessment showed a statistically significant correlation among these variables (rho = 0.26, *p* = 0.0186), although it demonstrated a weak association (Figure 3).

Additionally, the quantile regression model we utilized suggested that the association between keloid evolution time and treatment duration was defined as weak across all percentiles. Particularly, we observed that in the individuals with shorter treatment duration (25th percentile), each added month of keloid evolution enhanced the treatment duration by 0.054 months (95% CI: 0.033–0.073, *p* < 0.05), while at the median treatment duration (50th percentile), the calculated effect was still similar (0.056 months/month of keloid evolution [95% CI: 0.032–0.081, *p* < 0.05]). In individuals with longer treatment durations (75th percentile), the calculated enhancement per added month of keloid evolution was 0.057 months (95% CI −0.034 to 1.054).

## 4 Discussion

The first point to be highlighted in this study is the long follow‐up period, considering that only patients with high phototypes were selected for follow‐up. As recurrence often occurs in the first two years after treatment, our study ensured that follow‐up was long enough when analyzing patients [20].

The predominance of female patients (78.5%) is consistent with previous studies suggesting a higher predisposition in women for keloid development, possibly due to hormonal differences and immune response variations [21].

Pain was the only reported adverse effect (8.9%), but most patients experienced complete symptom relief (pain and itching) within four months of treatment initiation. Adverse effects were associated with a lower complete resolution rate (*p* = 0.019), indicating that patients experiencing pain might have lower adherence to treatment or poorer response. This is relevant considering that side effects are often a limitation to the continuation of treatment, especially pain and pruritus, so ensuring that these effects are transient can increase treatment adherence [22].

Our findings indicate that the ASAP algorithm is associated with significantly low recurrence rates (1.3%) and poses this sequential treatment as an effective option for keloid treatment, especially when considering patients with higher phototypes and the scarcity of studies in this specific population [23]. A previous comprehensive review noted that despite the higher incidence of keloids in individuals with darker skin types, this population is paradoxically underrepresented in research [23]. The current study, on the other hand, has involved mainly darker‐skinned individuals.

Complete resolution was achieved in 82.3% of patients, evidenced by improved erythema and total lesion flattening. This is relevant considering that keloids in ethnic pigmented skin are often a clinical challenge, and there is no established standardized method to treat these patients [24, 25].

High satisfaction (94.9%) among individuals with phototypes III–V is a noteworthy finding, given that post‐inflammatory hyperpigmentation and the risk of new keloid formation often restrict the use of aggressive technologies or surgical interventions in this population [25].

No association was found between gender and recurrence (*p* = 0.4855) or gender and complete resolution (*p* = 0.2863), suggesting that these outcomes are not influenced by patient sex. This may indicate that the ASAP method is robust and effective across female and male skin of color patients. This is especially relevant considering that studies have already demonstrated that gender may interfere with therapeutic success [26].

Patient satisfaction was significantly higher in cases of complete resolution (*p* = 0.000176), highlighting the importance of aesthetic and functional outcomes in treatment success perception. This emphasizes the importance of considering the patient's perception when analyzing treatment success, as the patient's and the observer's perceptions of the keloids are often different [27].

The treatment duration (mean of 18.95 months) did not correlate with complete resolution (*p* = 1.00), suggesting that duration alone is not a determining factor for success. The length of the treatment method did not negatively impact resolution rates.

Keloid location also did not influence resolution or recurrence (*p* > 0.05). This implies that the ASAP method is a therapeutic modality that maintains high resolution rates and low recurrence for keloids, regardless of their location. The logistic regression model failed to identify significant predictors of resolution, suggesting that the analyzed clinical and demographic variables may not be the main determinants of therapeutic success. This expands the idea that the ASAP method can guarantee good resolution rates regardless of factors such as age, gender, or keloid location. Some studies have shown that gender and age do not impact resolution rates, while location could be a factor in impacting resolution. These findings corroborate the idea that the ASAP method can be used in different patient profiles and keloids in different locations [21, 28].

Keloid evolution time showed a weak but significant correlation with treatment duration (*p* = 0.0186), indicating that older lesions may require longer interventions. This draws attention to the fact that older keloids are more difficult to treat, and therefore, early treatment could be a predictor of faster improvement. Studies have suggested that longer‐standing keloids may respond less favorably to treatment, highlighting the importance of timely intervention [29].

Corticosteroid infiltration before the initiation of the ASAP treatment was associated with longer treatment duration (*p* = 0.0022). Corticosteroid injections can lead to adverse effects, including telangiectasia, atrophy, steroid acne, pigmentary changes, necrosis, ulcerations, and systemic side effects. These complications may necessitate treatment strategy modification and could potentially complicate future therapeutic interventions [30]. Other possible explanations for an increased resistance to treatment in patients who had previously received steroid injections might be related to a well‐known variation in keloid phenotypes among individuals [31]. Mesenchymal fibroblast subpopulation heterogeneity has also been reported [32]. Keloids previously unresponsive to treatment with steroid injections might hold aggressive fibroblasts cell lines, contributing to resistant keloid phenotypes.

The findings of this study demonstrate a high success rate in keloid resolution in skin of color patients, combined with an extremely low recurrence rate in one of the largest samples of keloids treated using a sequential systematic method and followed for a longer period. Future studies with even larger samples should consider analyzing other modifying factors, such as an increased number of IPL sessions, to optimize clinical outcomes and reduce treatment duration.

## 5 Conclusion

The ASAP protocol represents a significant advancement in the treatment of keloids, delivering high patient satisfaction, low recurrence rates, and long‐term outcomes. This study, conducted on patients treated at least 19 months prior and followed up for nearly 10 years (123 months) without additional intervention, demonstrated a significantly lower recurrence in skin of color patients.

The ASAP algorithm, designed to assist dermatologists in systematically managing keloids, emphasizes the importance of sequential treatment and integration of diverse therapeutic modalities. The unique combination of occlusion, monthly sessions of 5‐FU and triamcinolone injections, and IPL therapy in a sequential method not only flattens keloids but also effectively addresses symptoms.

Despite its benefits, the prolonged treatment duration requires effort to ensure adherence, and its reliance on advanced dermatological expertise may pose challenges for non‐specialist providers. The single‐center design of this study highlights the need for future multicenter research with larger sample sizes to confirm these findings, compare with other treatment modalities and further establish the ASAP method as a standard of care for keloid management in skin of color patients.

By offering a non‐surgical, patient‐friendly approach with enduring outcomes, the ASAP protocol provides valuable insights for dermatologists and policymakers to optimize keloid treatment practices, ultimately enhancing patient care and satisfaction.

## Ethics Statement

We confirm that all material and methods were approved by the local Institutional Review Board and the Ethics Committee of the Medical Sciences Faculty of Minas Gerais and registered in Plataforma Brazil (#6.674.720—CAAE 65135922.0.3001.5134). This study was performed under the Code of Ethics of the World Medical Association (Declaration of Helsinki) and the Recommendations for the Conduct, Reporting, Editing and Publication of Scholarly Work in Medical Journals.

## Consent

Consent for the publication of recognizable patient photographs or other identifiable material was obtained by the authors and included at the time of article submission to the journal, stating that all patients gave consent with the understanding that this information may be publicly available.

## Conflicts of Interest

The authors declare no conflicts of interest.

## Data Availability

The data that support the findings of this study are available from the corresponding author upon reasonable request.
